# Genome-Wide MicroRNA Analysis of Peripheral Blood Mononuclear Cells Reveals Elevated miR-142-3p Expression as a Potential Biomarker for Secondary Syphilis

**DOI:** 10.1155/2021/5520053

**Published:** 2021-07-19

**Authors:** Zhusheng Yang, Xiumin Yang, Zhaoyan Gan, Liang Yuan, Wei Liu, Chaozeng Si

**Affiliations:** ^1^Department of Dermatology, Beijing Tongren Hospital, Capital Medical University, Beijing, China; ^2^Medical Laboratory, Beijing Tongren Hospital, Capital Medical University, Beijing, China; ^3^Department of Human Anatomy, Histology and Embryology, Institute of Basic Medical Sciences, Chinese Academy of Medical Sciences, China; ^4^Information Center, China-Japan Friendship Hospital, Beijing, China

## Abstract

**Background:**

*Treponema pallidum* subspecies *pallidum* (*T*. *pallidum*) infection induces significant immune responses, resulting in tissue damage. Gene expression plays an essential role in regulating the progression of syphilis infection. However, little is known about the regulatory role of microRNAs (miRNAs) in the immune response to *T*. *pallidum* infection. Here, we analyze the differential expression of miRNAs in peripheral blood mononuclear cells (PBMCs) between untreated secondary syphilis patients and healthy controls and study the correlation between miRNA expression and clinical features with bioinformatics.

**Methods:**

The expression profile of miRNAs was measured by microarray analysis in PBMCs of untreated secondary syphilis patients and healthy controls. Weighted Gene Coexpression Network Analysis (WGCNA) was used to construct the expression of miRNAs and the clinical data of secondary syphilis patients. Gene ontology (GO) and KEGG enrichment analyses were performed on target genes of miR-142-3p.

**Results:**

244 miRNAs exhibited at least 1.0-fold differential expression between secondary syphilis patients and healthy controls. The miRNAs were divided into three modules by WGCNA. The blue module was positively correlated with TPHA, TRUST, duration of disease, and erythema. And in the blue module, the expression of miR-142-3p was significantly higher in secondary syphilis patients than in healthy controls (*p* = 0.02), which is also close to the clinical features of secondary syphilis. GO and KEGG pathway analyses showed that these target genes of miR-142-3p are correlated with endocytosis and positive regulation of the apoptotic process.

**Conclusion:**

The elevated miR-142-3p expression in PBMCs may play an important role in the immune response to *T*. *pallidum* infection and may be a potential biomarker for secondary syphilis.

## 1. Introduction

Syphilis is a chronic classical sexually transmitted disease caused by the uncultivatable spirochaete *Treponema pallidum* subspecies *pallidum* (*T*. *pallidum*). Most of the symptoms and tissue damage related to syphilis are caused by activation of the host inflammatory and immune responses, suggesting that the host gene expression plays a vital role in regulating the progression of syphilis infection [1]. However, the mechanisms of this progression remain to be studied.

MicroRNAs (miRNAs) are a kind of small molecules with the transcriptional regulation activity of endogenous noncoding single-stranded RNAs [2, 3]. Recent studies have shown that miRNAs play an essential role in the human immune response to bacterial infection. For example, miRNAs regulate the immune response of hosts infected with pathogenic bacteria, such as *Helicobacter pylori*, *Salmonella*, *L*. *monocytogenes*, and *Mycobacterium* [4–7]. However, the potential regulatory role of miRNAs in the host antisyphilis immunity remains unclear.

To investigate the role of miRNAs in regulating the host antisyphilis immunity, we studied the expression profiles of miRNAs in peripheral blood mononuclear cells of patients with untreated secondary syphilis and healthy controls by microarray. Then, we examined the correlation between miRNA expression and clinical features with bioinformatics.

## 2. Materials and Methods

### 2.1. Participants

Six untreated secondary syphilis patients and six healthy controls were first recruited in the microRNA array study. Another 21 untreated secondary syphilis patients and 14 healthy controls were recruited for the quantitative RT-PCR (qRT-PCR) assay for the validation of the microarray data (Table 1). The diagnosis of secondary syphilis was based on the medical history and compatible skin or mucosal lesions, a reactive nontreponemal test (toluidine red unheated serum test (TRUST)), and a positive confirmatory treponemal test (*Treponema pallidum* particle hemagglutination assay (TPHA)). Patients were excluded if they (1) were known to be HIV-positive, (2) had serologic evidence of current or prior infection with hepatitis B or hepatitis C, (3) were receiving anti-inflammatory or immunosuppressive medications, (4) had recently used antibiotics, and (5) had a history of chronic dermatitis or other underlying acute or chronic disease. This study was approved by the Institutional Ethics Committee of Beijing Tongren Hospital, Capital Medical University (TRECKY2015-031), in accordance with the Helsinki Declaration amended in 2008. All participants signed informed consent.

### 2.2. PBMC Preparation and RNA Extraction

PBMCs were extracted immediately after the collection of whole blood using a standard procedure of Ficoll density gradient centrifugation, performed in accordance with the manufacturer's instructions (Solarbio, Beijing, China). Total RNA (including miRNAs) was isolated from human PBMCs using the miRNeasy mini kit (Qiagen, Valencia, CA, USA) following the manufacturer's protocol. RNA concentration, including OD260/280 and OD260/230 values, was determined by using a spectrophotometer (NanoDrop 2000, Thermo Scientific, USA). The results of RNA electrophoresis showed that the quality of RNA extraction was good.

### 2.3. Microarray Analysis, Data Collection, and Preprocessing

For microRNA expression analysis, the Affymetrix GeneChip miRNA 2.0 array was used according to the manufacturer's instructions by GeneTech (Shanghai) Company Limited. The chip quality control report generated by Expression Console Software of Affymetrix Company includes quality control results, detection of spikes in the probe, and evaluation signal values. Data were normalized, and then miRNA differential expression analysis was conducted using the R package limma [8]. Differentially expressed miRNAs (DEmiRNAs) were identified by comparing secondary syphilis patients with healthy controls. Adjusted *p* value < 0.05 and ∣log2 fold change (log2FC) | >1 were set as the thresholds for identifying DEmiRNAs. The microarray data are available from the NCBI Gene Expression Omnibus database (GEO: GSE156421).

### 2.4. Construction of the WGCNA Coexpression Network

The Weighted Gene Coexpression Network Analysis (WGCNA) method [9] was used to construct the coexpression network based on the expression of miRNAs. An appropriate soft threshold power *β* = 6 was selected by using the integrated function (pick soft threshold) in the WGCNA package. With this soft threshold power, the coexpression similarity was raised to achieve scale-free topology. We used module-trait relationships (MTRs) to determine the significant correlation between module eigengenes and clinical traits of syphilis patients. For WGCNA, we evaluated the Gene Significance (GS) and Module Membership (MM). GS is the absolute value of the correlation between a specific gene and a trait; MM is the correlation between the module eigengene and the gene expression profile. By analysis of GS and MM, we identified miRNAs that showed significant MM and high GS for syphilis.

### 2.5. Quantitative Real-Time PCR

Take 300 ng of the total RNA extracted from 14 healthy controls and 21 secondary syphilis patients as the template and reverse transcribe to generate the cDNA. The expression of miR-142-3p was detected with U6 as the internal parameter. Quantitative real-time polymerase chain reactions (qRT-PCR) were incubated in a 96-well plate at 95°C for 10 min, followed by 40 cycles of 95°C for 15 s and 60°C for 1 min. The forward primer of miR-142-3p was UGUAGUGUUUCCUACUUUAUGGA.

### 2.6. Target Gene Prediction and Pathway Analysis

We used three public databases to predict the target genes of miR-142-3p: miRDB [10], miRWalk [11], and starBase [12]. By using the intersected genes, pathway analysis of GO and KEGG was produced using clusterProfiler [13]. The statistical cutoff value was *p* < 0.05.

### 2.7. Statistical Analysis

Numerical data were compared using Student's *t*-test, and categorical data were compared using the Fisher exact test. All statistical analyses were performed by R 3.5.0. A *p* value < 0.05 was considered statistically significant.

## 3. Results

### 3.1. Differentially Expressed miRNAs in PBMCs of Patients with Secondary Syphilis

**Figure 1:**
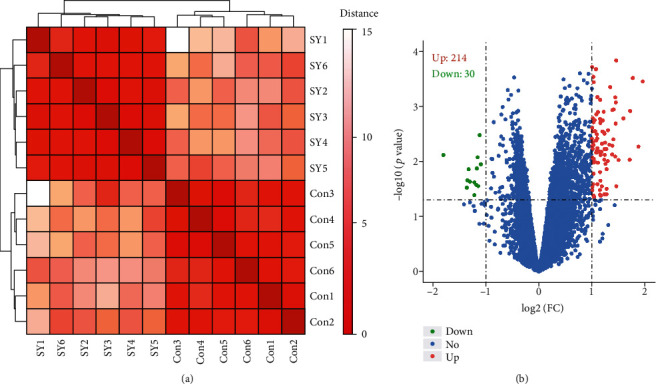
Correlation distance heatmap and differential expression of miRNAs between the secondary syphilis and healthy control groups. (a) The correlation distance heatmap of secondary syphilis (*n* = 6) and healthy control samples (*n* = 6). (b) Volcano plot of differentially expressed miRNAs contrasting secondary syphilis and healthy control samples. The cutoff value for DEmiRNA significance was*p* value < 0.05, and the absolute value of the ∣log2 fold change (log2FC) | >1. The *y*-axis displays the −log10(*p* value) for each miRNA, while the *x*-axis displays the log2 fold change. Green dots represent downregulated miRNAs, red dots represent upregulated miRNAs, and blue dots indicate miRNA nonsignificance in our results.

We selected 6 untreated secondary syphilis patients and 6 healthy controls for miRNA chip analysis. By calculating the distance matrix using the Euclidean distance algorithm, we got the clustering heatmap of samples. The results of the clustering heatmap based on the miRNA expression matrix showed that secondary syphilis patients and healthy controls were clustered into two different groups (Figure 1(a)). DEmiRNAs between healthy controls and secondary syphilis patients were determined according to the microRNA expression value of the microarray (Supplementary table 1). The results also showed that the individuals in the two groups showed the right consistency. The volcano plot of differentially expressed miRNA results showed that 30 downregulated miRNAs showed substantial differences in green dots, 214 upregulated miRNAs showed significant differences in red dots, and upregulated miRNAs were more than downregulated miRNAs (Figure 1(b)).

### 3.2. Differentially Expressed miRNA Cluster Is Related to Clinical Traits

**Figure 2:**
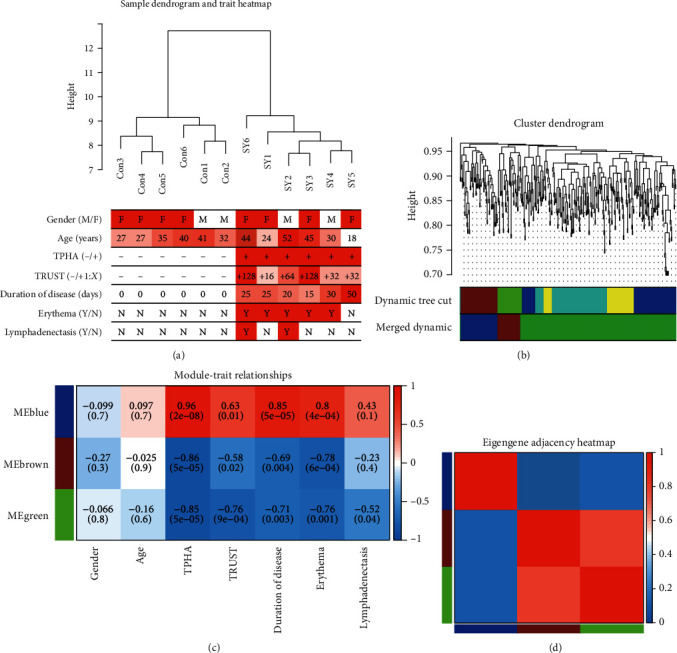
WGCNA results based on the expression of miRNAs. (a) Sample dendrogram and trait heatmap. The *y*-axis represents the distance of samples in the secondary syphilis and healthy control groups. (b) Cluster dendrogram of miRNAs and three modules were merged in a dynamic graph. (c) The correlation between microRNA modules and clinical traits. Red means a positive correlation, and blue means a negative correlation. (d) Eigengene adjacency heatmap of three merged modules.

To show the information of the individuals, we selected more clearly the relationship between the individuals of the healthy control group and the individuals of the secondary syphilis group, which is represented by the biological tree diagram, and their clinical traits include gender, age, TPHA, TRUST, duration of disease, erythema, and lymphadenectasis, which were displayed by the sample dendrogram and trait heatmap (Figure 2(a)). Using the WGCNA method to analyze miRNAs, we can see that the miRNAs can be divided into five modules according to their expression patterns, represented by five color blocks (Figure 2(b)), and then further classified into three modules, which are represented by brown, green and blue, respectively. Blue blocks contain the most significant number of miRNAs. To better understand the relationship between miRNAs in the three modules and clinical traits of secondary syphilis patients, we conducted a correlation analysis. The results showed that the blue module was positively correlated with TPHA, TRUST, duration of disease, and erythema, and the *p* values were most significant. The brown and green modules were negatively correlated with TPHA, TRUST, duration of disease, and erythema (Figure 2(c)). The eigengene adjacency heatmap analysis of the modules also reflects the differences between the blue module and the brown and green modules (Figure 2(d)).

### 3.3. The Target Genes of miR-142-3p May Be Involved in the Development of Secondary Syphilis Immunity

**Figure 3:**
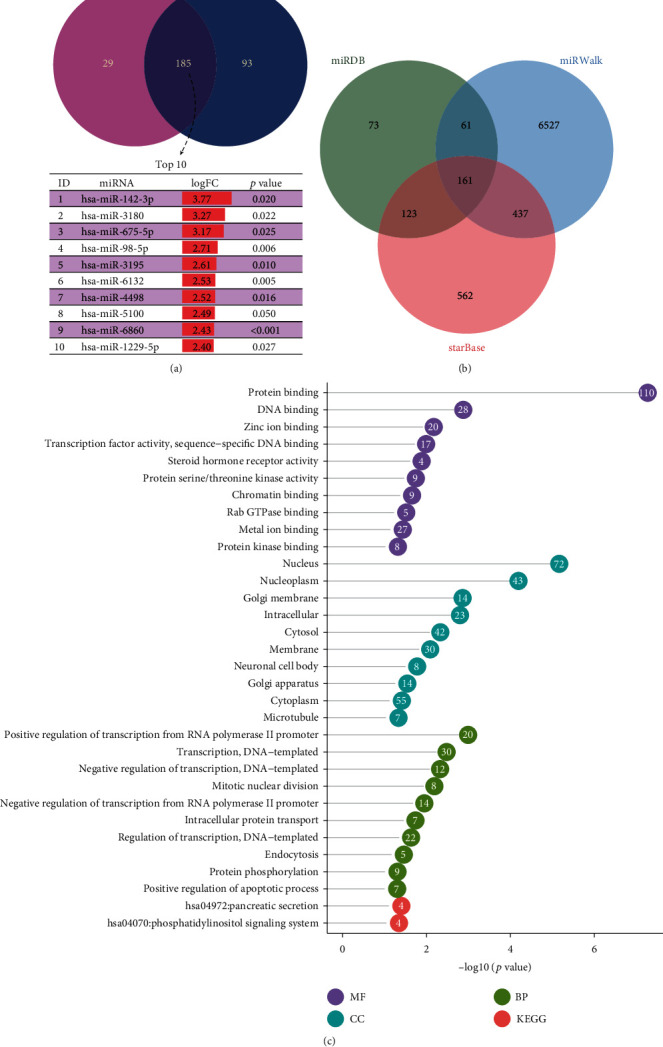
Target gene prediction and GO and KEGG pathway analyses. (a) By overlapping upregulated miRNAs and MEblue module miRNAs, 185 miRNAs were figured out, and the top 10 most significant miRNAs were listed. (b) Target genes of miR-142-3p were predicted based on three databases: miRDB, miRWalk, and starBase, and 161 common genes were obtained by the Venn diagram. (c) Top 10 of each GO term, including the biological process (BP), cellular component (CC), molecular function (MF), and KEGG pathways, were all enriched. The *x*-axis represents the −log10(*p* value), and the number of genes enriched in each pathway was also marked on each circle.

**Figure 4:**
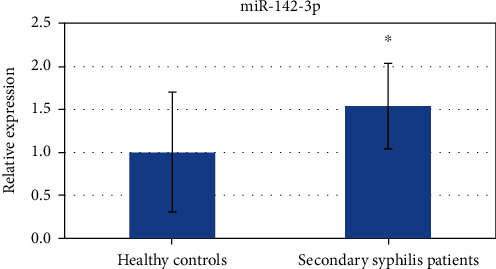
qPCR results of miR-142-3p in healthy controls and secondary syphilis patients. The expression levels of the healthy controls (14 cases) and secondary syphilis patients (21 cases) were shown by blue bars, respectively, by the results of the 2^-*ΔΔ*CT^ method with three biological repeats. A significant difference was detected in the two groups (^∗^*p* < 0.05).

**Figure 5:**
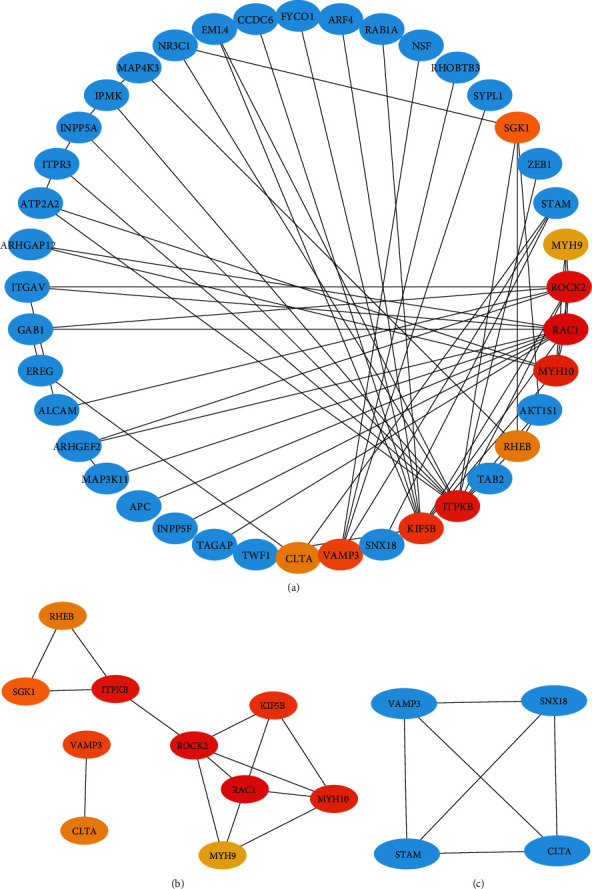
The PPI network of 161 target genes of miR-142-3p. (a) The PPI network of target genes of miR-142-3p. (b, c) The subnetworks of the PPI network analyzed by MCODE using Cytoscape.

To study the function of miRNAs in secondary syphilis, we overlapped the miRNAs in the blue module with the upregulated miRNAs and got 185 common miRNAs. The most significant fold change in the 185 miRNAs was miR-142-3p (Figure 3(a)). To validate the function of miR-142-3p in syphilis, we selected 14 healthy controls and 21 secondary syphilis patients for qPCR conformation. The results indicated that the expression level of miR-142-3p in untreated secondary syphilis patients was higher than that in healthy controls (*p* = 0.038) and is consistent with that of the microRNA array (Figure 4). The target genes of miR-142-3p were predicted based on three databases: miRDB (http://mirdb.org/), miRWalk (http://mirwalk.umm.uni-heidelberg.de/), and starBase (http://starbase.sysu.edu.cn/index.php). We draw a Venn diagram for the target genes predicted by each database. The Venn diagram shows 161 target genes predicted by the three websites (Figure 3(b)). Then, we performed GO and KEGG enrichment analyses (Supplementary table 2). Molecular function (MF) analysis showed that these target genes are correlated with protein binding, DNA binding, zinc ion binding, etc. Cellular component (CC) analysis showed that these target genes are correlated with the nucleus, nucleoplasm, Golgi membrane, etc. Biological process (BP) analysis showed that these target genes are correlated with positive regulation of transcription from the RNA polymerase II promoter, DNA-templated transcription, mitotic nuclear division, DNA-templated negative regulation of transcription, mitotic nuclear division, negative regulation of transcription from the RNA polymerase II promoter, intracellular protein transport, DNA-templated regulation of transcription, endocytosis, protein phosphorylation, and positive regulation of the apoptotic process. KEGG pathway analysis showed that these target genes are correlated with pancreatic secretion and the phosphatidylinositol signaling system (Figure 3(c)). The PPI (protein-protein interaction) network was also analyzed. The results of the PPI network showed that most target genes interacted (Figure 5(a)). And the subnetworks of the PPI network were analyzed by MCODE in Cytoscape (Figures 5(b) and 5(c)).

### 3.4. Differential Expression of miR-142-3p Could Be a Potential Biomarker for Secondary Syphilis

**Figure 6:**
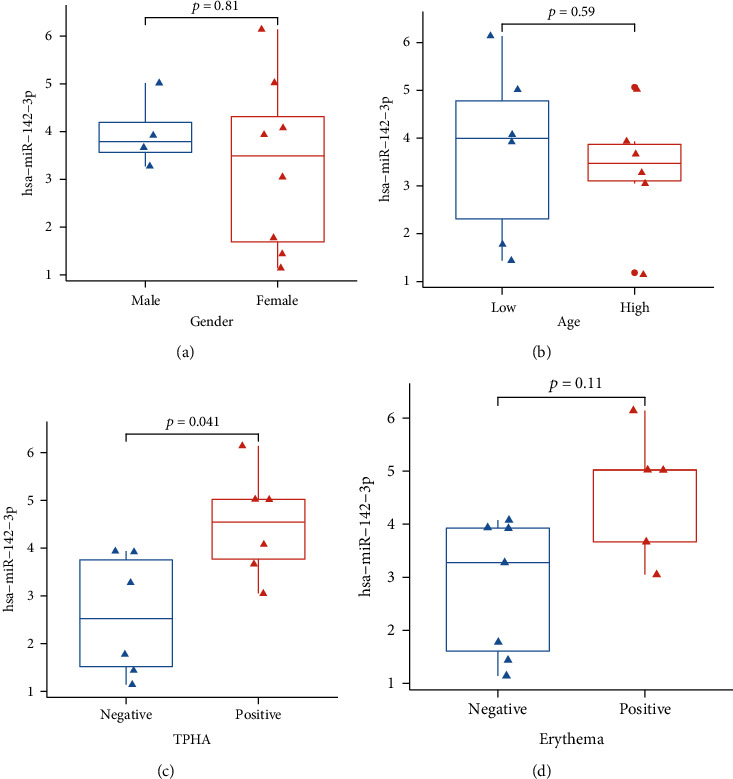
The expression of miR-142-3p in different clinical phenotypic traits. (a) miR-142-3p expression between males (*n* = 4) and females (*n* = 8). (b) miR-142-3p expression in different age groups. (c) miR-142-3p expression between different TPHA groups. Negative (*n* = 6), positive (*n* = 6). (d) miR-142-3p expression between different erythema groups. Negative (*n* = 7), positive (*n* = 5).

To further understand the expression level of miR-142-3p in the samples, we analyzed the clinical traits such as gender, age, TPHA, and erythema. Among the gender traits, the expression level of miR-142-3p in the male group was higher than that in the female group, but there was no significant difference (Figure 6(a)). Among the age traits, the expression level of miR-142-3p in the elderly group was higher than that in the younger group, but there was no significant difference (Figure 6(b)). Among the TPHA traits, the expression level of miR-142-3p in the TPHA-negative group was significantly lower than that in the TPHA-positive group (*p* < 0.05) (Figure 6(c)). Among the erythema traits, the expression level of miR-142-3p in the erythema-negative group was lower than that in the erythema-positive group, but there was no significant difference between the two groups (Figure 6(d)).

## 4. Discussion

As a class of small molecules that can regulate gene expression, miRNAs play an important role in the regulation of the host innate and adaptive immune responses to pathogen infection, but little is known about the regulation of miRNAs in the host antisyphilis immunity. By analyzing the differential expression of miRNAs in PBMCs between untreated secondary syphilis patients and healthy controls, this study revealed a variety of miRNAs involved in the host antisyphilis immune response, and further analysis showed that the differentially expressed miR-142-3p may play an important regulatory role in the host immune response to *T*. *pallidum* infection in secondary syphilis.

Prior to our study, only limited studies have been carried out on the expression profile of miRNAs in peripheral blood of syphilis. In our study, 244 differentially expressed miRNAs were found in secondary syphilis patients compared with healthy controls. According to the microarray analysis, 214 miRNAs in untreated secondary syphilis patients were upregulated relative to those in healthy controls, and 30 miRNAs were downregulated (Figure 1(b)). However, the differential expression of miRNAs is different between the study of Huang et al. [14] and ours. The reason for this difference may be caused by the difference in the selection of subjects (syphilis patients). Huang et al.'s study compared 6 healthy controls with 6 untreated syphilis patients (including 3 primary and 3 secondary syphilis patients), while our study compared 6 healthy controls with 6 untreated secondary syphilis patients. The difference between the two results further suggests that the differences in miRNA expression may play an important role in the progression of syphilis. This is worthy of further research.

miR-142-3p is the most significant among the top 10 DEmiRNAs in PBMCs (Figure 3(a)). Further pathway analysis of predicted target genes for miR-142-3p was involved in endocytosis, positive regulation of transcription, mitotic nuclear division, protein phosphorylation, positive regulation of the apoptotic process, etc. (Figure 3(c)). Phagocytosis of dendritic cells (DCs), involved in driving the cellular immune processes, is a characteristic of syphilis infection [15]. After pathogen sensitization in the host was eventually triggered, *T*. *pallidum* was engulfed by DCs via both coiling phagocytosis and conventional phagocytosis and was delivered to membrane-bound vacuoles [15]. In addition, the phagocytosis of macrophages plays an important role in the subsequent immune clearance of *T*. *pallidum* organisms [16]. Interestingly, previous research indicated that overexpression of miR-142-3p in human monocyte-derived macrophages and DCs significantly attenuated the phagocytosis of *Escherichia coli* and *Staphylococcus aureus*, as well as the secretion of inflammatory mediators, including TNF-*α*, IL-6, and IL-12p40 [17–19]. Based on these findings, we speculated that the increased expression of miR-142-3p may have suppressed the phagocytosis of DCs and macrophages in secondary syphilis patients, thus contributing to the development of *T*. *pallidum* infection.

Clarifying the endogenous regulatory mechanism of miRNAs on gene expression of the immune system has made us not only have a deeper understanding of the pathogenesis of many infectious diseases, such as syphilis [20, 21], but also find biomarkers to evaluate the efficacy and predict the prognosis. In our study, the elevated expression of miR-142-3p in PBMCs was related to the clinical traits of secondary syphilis (Figure 6), suggesting that it could serve as a potential marker of the disease. At the same time, much more research should be done to reveal the function of miR-142-3p in specific immune cell types in the near future.

## 5. Conclusion

In summary, our research found that the elevated miR-142-3p expression in PBMCs may play an important role in the immune response to *T*. *pallidum* infection. It could be a potential biomarker for secondary syphilis, which may make a foundation for further application in the diagnosis, prognosis, and specific treatment targets.

## Figures and Tables

**Table 1:** Demographic characteristics of secondary syphilis patients and healthy controls.

	Microarray study	Validation study
*Secondary syphilis group*		
Number of participants	6	21
Sex (male/female)	2/4	10/11
Age (years, average; range)	36 (18-52)	34 (27-42)
Duration of disease (days, average; range)	28 (14-50)	26 (10-50)
TPHA (positive/negative)	6/0	21/0
TRUST titer (positive/negative)	6/0	21/0
TRUST titer 1 : 128	2	7
TRUST titer 1 : 64	1	3
TRUST titer 1 : 32	2	3
TRUST titer 1 : 16	1	6
TRUST titer 1 : 8	0	2
Skin lesions and symptoms		
Roseola	1	6
Plaques on palms and/or soles	2	7
Moth-eaten alopecia	1	5
Condylomata lata	1	3
Mild flu-like symptoms (i.e., headache, myalgias)	2	8
Adenopathy	2	10
*Healthy control group*		
Number of participants	6	14
Sex (male/female)	2/4	6/8
Age (years, average; range)	34 (27-42)	35 (23-47)
TPHA (positive/negative)	0/6	0/14
TRUST titer (positive/negative)	0/6	0/14
Skin lesions and symptoms	0	0

TPHA: *Treponema pallidum* particle hemagglutination assay; TRUST: toluidine red unheated serum test.

## Data Availability

The datasets used or analyzed during the current study are available from the corresponding author.

## Supplementary Materials

Supplementary 1 Supplementary table 1: differentially expressed miRNAs in PBMCs of patients with secondary syphilis.

Supplementary 2 Supplementary table 2: GO and KEGG enrichment analyses.
