# Supplementary material for: Genome-Wide MicroRNA Analysis of Peripheral Blood Mononuclear Cells Reveals Elevated miR-142-3p Expression as a Potential Biomarker for Secondary Syphilis
Source: Biomed Res Int. 2021 Jul 19;2021:5520053. doi: 10.1155/2021/5520053 (PMC8317471; doi:10.1155/2021/5520053)
Supplement: Supplementary 1 — Supplementary table 1: differentially expressed miRNAs in PBMCs of patients with secondary syphilis. [file 5520053.f1.pdf]

Supplementary table 1 Differential expression miRNAs between healthy controls and secondary syphilis patients (MicroRNAs chip analysis was performed in 6 healthy controls and 6 untreated secondary syphilis patients).

Transcript Cluster ID	Transcript ID(Array Design)	Accession	Sample Bi-weight Avg Signal (log2)	Control Bi-weight Avg Signal (log2)	Sample Standard Deviation	Control Standard Deviation	Fold Change (linear) (Sample [illegible]	ANOVA p-value (Sample vs. Control)	FDR p-value (Sample vs. Control)
20501285	hsa-miR-326	MIMAT0000756	3.62	1.66	0.96	0.88	3.9	0.03572	0.783209
20500750	hsa-miR-142-3p	MIMAT0000434	4.49	2.57	1.11	1.28	3.77	0.019752	0.783209
20517745	hsa-miR-4286	MIMAT0016916	4.2	2.44	0.69	1.25	3.39	0.031679	0.783209
20518425	hsa-miR-3180	MIMAT0018178	3.27	1.56	0.73	0.99	3.27	0.021688	0.783209
20505608	hsa-miR-675-5p	MIMAT0004284	4.31	2.64	0.89	0.76	3.17	0.024799	0.783209
20525743	hsa-miR-6891-5p	MIMAT0027682	4.57	3.03	0.4	0.96	2.9	0.018215	0.783209
20500179	hsa-miR-98-5p	MIMAT0000096	4.6	3.16	0.43	1.09	2.71	0.006222	0.687402
20515637	hsa-miR-3195	MIMAT0015079	4.05	2.67	0.7	0.78	2.61	0.010002	0.72094
20524053	hsa-miR-6132	MIMAT0024616	4.12	2.78	0.48	0.54	2.53	0.004595	0.64955
20517744	hsa-miR-4284	MIMAT0016915	4.71	3.37	0.79	0.7	2.53	0.013327	0.783209
20518893	hsa-miR-4498	MIMAT0019033	4.08	2.74	0.6	0.63	2.52	0.015736	0.783209
20521785	hsa-miR-5100	MIMAT0022259	5.63	4.31	0.72	0.84	2.49	0.049759	0.814413
20525683	hsa-miR-6860	MIMAT0027622	3.28	2	0.23	0.33	2.43	0.000016	0.108216
20506776	hsa-miR-1229-5p	MIMAT0022942	3.89	2.63	0.43	0.77	2.4	0.027029	0.783209
20518783	hsa-miR-378e	MIMAT0018927	2.86	1.61	0.84	0.41	2.37	0.030974	0.783209
20506779	hsa-miR-1231	MIMAT0005586	3.2	1.98	0.49	0.56	2.34	0.016718	0.783209
20525446	hsa-miR-6742-5p	MIMAT0027385	2.22	1.03	0.47	0.36	2.29	0.002179	0.638484

20500143	hsa-miR-22-5p	MIMAT0004495	4.81	3.63	0.37	0.9	2.27	0.009742	0.72094
20525023	hsa-miR-6511a-5p	MIMAT0025478	3.52	2.35	0.64	0.59	2.26	0.013829	0.783209
20518935	hsa-miR-4534	MIMAT0019073	4.31	3.17	0.45	0.9	2.2	0.024249	0.783209
20525383	hsa-miR-6716-5p	MIMAT0025844	2.71	1.59	0.46	0.34	2.17	0.00083	0.411497
20503885	hsa-miR-504-3p	MIMAT0026612	3.26	2.14	0.42	0.39	2.17	0.001215	0.473808
20518825	hsa-miR-4448	MIMAT0018967	2.04	0.92	0.64	0.37	2.16	0.01403	0.783209
20518721	hsa-miR-642b-3p	MIMAT0018444	3.47	2.37	0.6	0.69	2.15	0.008331	0.687402
20504574	hsa-miR-454-3p	MIMAT0003885	4.47	3.39	0.34	0.68	2.12	0.004113	0.64955
20525657	hsa-miR-6848-5p	MIMAT0027596	3.23	2.16	0.41	0.79	2.11	0.025499	0.783209
20506837	hsa-miR-1246	MIMAT0005898	3.72	2.65	0.72	0.7	2.09	0.022294	0.783209
20529773	hsa-miR-8063	MIMAT0030990	2.54	1.49	0.41	0.42	2.08	0.009028	0.712666
20529568	hsa-miR-7977	MIMAT0031180	6.39	5.38	0.55	0.6	2.01	0.023619	0.783209
20526861	hsa-miR-7150	MIMAT0028211	4.28	3.29	0.39	0.74	1.98	0.030083	0.783209
20512262	hsa-miR-2277-3p	MIMAT0011777	2.59	1.63	0.29	0.3	1.95	0.000195	0.260306
20503102	hsa-miR-484	MIMAT0002174	5.5	4.53	0.35	0.66	1.95	0.021597	0.783209
20532829	ENSG00000201009	ENSG00000201	5.27	4.33	0.77	0.64	1.92	0.046865	0.802171
20538187	U46	U46	5.27	4.33	0.77	0.64	1.92	0.046865	0.802171
20529783	hsa-miR-8073	MIMAT0031000	2.11	1.18	0.43	0.28	1.9	0.003145	0.64955
20525701	hsa-miR-6870-5p	MIMAT0027640	2.15	1.27	0.36	0.38	1.84	0.001073	0.44465
20525721	hsa-miR-6880-5p	MIMAT0027660	4.63	3.75	0.17	0.85	1.84	0.043985	0.801082
20525525	hsa-miR-6782-5p	MIMAT0027464	3.02	2.15	0.51	0.36	1.83	0.004344	0.64955

20529781	hsa-miR-8071	MIMAT0030998	2.48	1.6	0.39	0.53	1.83	0.041156	0.79565
20501159	hsa-miR-29c-5p	MIMAT0004673	2.5	1.65	0.69	0.46	1.81	0.032004	0.783209
20525523	hsa-miR-6781-5p	MIMAT0027462	2.44	1.61	0.54	0.33	1.78	0.015557	0.783209
20515560	hsa-miR-3147	MIMAT0015019	2.84	2.01	0.51	0.82	1.77	0.041064	0.79565
20519439	hsa-miR-4655-5p	MIMAT0019721	3.5	2.69	0.32	0.41	1.76	0.000941	0.416034
20518826	hsa-miR-4449	MIMAT0018968	3.52	2.71	0.31	0.59	1.75	0.013577	0.783209
20519609	hsa-miR-4750-5p	MIMAT0019887	3.98	3.19	0.47	0.69	1.72	0.035613	0.783209
20521810	hsa-miR-664b-5p	MIMAT0022271	2.05	1.29	0.34	0.24	1.7	0.003842	0.64955
20515624	hsa-miR-3188	MIMAT0015070	2.13	1.38	0.45	0.27	1.68	0.007948	0.687402
20500435	hsa-miR-7-5p	MIMAT0000252	2.13	1.42	0.58	0.23	1.63	0.01169	0.775194
20538274	U94	U94	2.04	1.36	0.48	0.37	1.6	0.033644	0.783209
20509226	hsa-miR-1909-5p	MIMAT0007882	2.33	1.65	0.5	0.25	1.6	0.036084	0.783209
20500461	hsa-miR-204-3p	MIMAT0022693	2.45	1.79	0.38	0.57	1.59	0.028842	0.783209
20518865	hsa-miR-4478	MIMAT0019006	1.95	1.28	0.63	0.32	1.59	0.043403	0.801082
20536978	hsa-mir-4740	MI0017378	1.8	1.14	0.25	0.34	1.58	0.005339	0.680859
20519556	hsa-miR-4722-3p	MIMAT0019837	2.65	2	0.53	0.26	1.57	0.028308	0.783209
20515540	hsa-miR-3136-5p	MIMAT0015003	1.71	1.06	0.29	0.27	1.56	0.006814	0.687402
20505609	hsa-miR-675-3p	MIMAT0006790	1.98	1.34	0.33	0.35	1.56	0.010607	0.73267
20519595	hsa-miR-4743-5p	MIMAT0019874	3.77	3.14	0.41	0.55	1.55	0.01835	0.783209
20501177	hsa-miR-99b-3p	MIMAT0004678	2.27	1.64	0.83	0.18	1.55	0.020248	0.783209
20500393	hsa-miR-196a-5p	MIMAT0000226	1.72	1.09	0.69	0.14	1.55	0.030158	0.783209

20515573	hsa-miR-3154	MIMAT0015028	1.88	1.25	0.35	0.25	1.54	0.005262	0.680859
20506785	hsa-miR-1236-5p	MIMAT0022945	1.55	0.93	0.36	0.3	1.54	0.016813	0.783209
20515572	hsa-miR-3074-3p	MIMAT0015027	1.67	1.05	0.78	0.28	1.54	0.034959	0.783209
20515563	hsa-miR-3149	MIMAT0015022	1.79	1.18	0.46	0.23	1.53	0.007558	0.687402
20525453	hsa-miR-6746-5p	MIMAT0027392	2.01	1.39	0.34	0.39	1.53	0.015704	0.783209
20517832	hsa-miR-23c	MIMAT0018000	3.2	2.59	0.42	0.66	1.53	0.038822	0.792084
20532686	ACA4	ACA4	3.09	2.51	0.43	0.16	1.5	0.004351	0.64955
20519570	hsa-miR-4728-5p	MIMAT0019849	5.05	4.47	0.25	0.39	1.5	0.047058	0.802171
20525553	hsa-miR-6796-5p	MIMAT0027492	1.92	1.37	0.43	0.2	1.47	0.013145	0.783209
20518823	hsa-miR-4446-3p	MIMAT0018965	3.59	3.03	0.61	0.7	1.47	0.046085	0.802069
20538178	U42B	U42B	3.05	2.5	0.58	0.24	1.46	0.014132	0.783209
20525497	hsa-miR-6768-5p	MIMAT0027436	2.05	1.5	0.39	0.3	1.46	0.037424	0.783209
20519655	hsa-miR-4776-5p	MIMAT0019932	2.28	1.73	0.57	0.52	1.46	0.040649	0.79565
20504284	hsa-miR-563	MIMAT0003227	1.89	1.35	0.31	0.17	1.45	0.003936	0.64955
20517723	hsa-miR-2355-5p	MIMAT0016895	1.34	0.8	0.35	0.17	1.45	0.010841	0.741066
20517692	hsa-miR-4312	MIMAT0016864	1.46	0.93	0.46	0.1	1.45	0.015706	0.783209
20501155	hsa-miR-194-3p	MIMAT0004671	1.54	1	0.3	0.2	1.45	0.019279	0.783209
20501183	hsa-miR-30e-3p	MIMAT0000693	6.46	5.94	0.24	0.28	1.43	0.006386	0.687402
20538179	U42B	U42B	5.25	4.74	0.61	0.25	1.43	0.017591	0.783209
20517749	hsa-miR-4289	MIMAT0016920	1.73	1.21	0.24	0.31	1.43	0.037695	0.783209
20535798	hsa-mir-1229	MI0006319	1.61	1.1	0.22	0.13	1.42	0.000869	0.411497

20525735	hsa-miR-6887-5p	MIMAT0027674	1.82	1.32	0.48	0.19	1.42	0.020113	0.783209
20515623	hsa-miR-3187-3p	MIMAT0015069	1.99	1.49	0.25	0.22	1.41	0.013919	0.783209
20517709	hsa-miR-4260	MIMAT0016881	1.4	0.92	0.19	0.24	1.4	0.006049	0.687402
20505789	hsa-miR-885-5p	MIMAT0004947	1.87	1.38	0.36	0.3	1.4	0.028597	0.783209
20534154	ENSG00000265013	ENSG0000026[illegible]	2.43	1.95	0.14	0.17	1.39	0.000393	0.260306
20534164	ENSG00000265634	ENSG0000026[illegible]	2.43	1.95	0.14	0.17	1.39	0.000393	0.260306
20534166	ENSG00000265663	ENSG0000026[illegible]	2.43	1.95	0.14	0.17	1.39	0.000393	0.260306
20534175	ENSG00000265926	ENSG0000026[illegible]	2.43	1.95	0.14	0.17	1.39	0.000393	0.260306
20534178	ENSG00000266132	ENSG0000026[illegible]	2.43	1.95	0.14	0.17	1.39	0.000393	0.260306
20534184	ENSG00000266672	ENSG0000026[illegible]	2.43	1.95	0.14	0.17	1.39	0.000393	0.260306
20511560	hsa-miR-2116-5p	MIMAT0011160	1.36	0.88	0.28	0.24	1.39	0.008501	0.687402
20504345	hsa-miR-605-5p	MIMAT0003273	1.8	1.32	0.26	0.25	1.39	0.011046	0.742701
20537009	hsa-mir-4767	MI0017408	1.94	1.47	0.29	0.16	1.39	0.018804	0.783209
20532718	ACA6	ACA6	4.51	4.05	0.18	0.35	1.38	0.012005	0.783209
20524052	hsa-miR-6131	MIMAT0024615	1.62	1.16	0.17	0.31	1.38	0.018906	0.783209
20519555	hsa-miR-4722-5p	MIMAT0019836	1.47	1	0.38	0.15	1.38	0.025758	0.783209
20506847	hsa-miR-1254	MIMAT0005905	1.6	1.14	0.34	0.28	1.38	0.031717	0.783209
20537087	hsa-mir-5095	MI0018001	4.14	3.68	0.25	0.43	1.38	0.031996	0.783209
20533151	ENSG00000221164	ENSG0000022[illegible]	1.39	0.93	0.18	0.11	1.37	0.000522	0.301727
20519676	hsa-miR-2467-3p	MIMAT0019953	1.92	1.46	0.38	0.29	1.37	0.049253	0.814413
20500154	hsa-miR-26b-5p	MIMAT0000083	7.81	7.37	0.18	0.32	1.36	0.004808	0.64955

20525615	hsa-miR-6827-5p	MIMAT0027554	1.31	0.87	0.2	0.3	1.36	0.030136	0.783209
20500141	hsa-miR-21-5p	MIMAT0000076	9.57	9.14	0.2	0.31	1.35	0.007067	0.687402
20500741	hsa-miR-135a-3p	MIMAT0004595	1.71	1.28	0.28	0.14	1.35	0.008216	0.687402
20536514	hsa-mir-500b	MI0015903	2.32	1.89	0.6	0.39	1.35	0.030222	0.783209
20536557	hsa-mir-3653	MI0016053	3.49	3.06	0.53	0.17	1.35	0.036902	0.783209
20538115	SNORD125	SNORD125	3.49	3.06	0.53	0.17	1.35	0.036902	0.783209
20538049	hsa-mir-8089	MI0025925	1.44	1.01	0.33	0.15	1.34	0.020573	0.783209
20536319	hsa-mir-3199-1	MI0014247	1.18	0.76	0.22	0.25	1.34	0.037038	0.783209
20536322	hsa-mir-3199-2	MI0014248	1.18	0.76	0.22	0.25	1.34	0.037038	0.783209
20536654	hsa-mir-550b-1	MI0016686	1.79	1.37	0.31	0.14	1.33	0.026067	0.783209
20536655	hsa-mir-550b-2	MI0016687	1.79	1.37	0.31	0.14	1.33	0.026067	0.783209
20519488	hsa-miR-4684-3p	MIMAT0019770	1.69	1.28	0.43	0.32	1.33	0.037117	0.783209
20519533	hsa-miR-4710	MIMAT0019815	3.56	3.15	0.29	0.21	1.33	0.040642	0.79565
20520351	hsa-miR-1273g-3p	MIMAT0022742	10.73	10.33	0.31	0.26	1.33	0.046005	0.802069
20505801	hsa-miR-760	MIMAT0004957	2.11	1.71	0.1	0.39	1.32	0.033276	0.783209
20500168	hsa-miR-33a-5p	MIMAT0000091	1.31	0.91	0.34	0.19	1.32	0.036777	0.783209
20506822	hsa-miR-1294	MIMAT0005884	1.75	1.34	0.23	0.34	1.32	0.046018	0.802069
20518901	hsa-miR-4505	MIMAT0019041	5.98	5.58	0.19	0.24	1.31	0.009344	0.720486
20537988	hsa-mir-7845	MI0025515	1.73	1.33	0.2	0.19	1.31	0.016071	0.783209
20535216	hsa-mir-507	MI0003194	1.27	0.9	0.15	0.22	1.3	0.021918	0.783209
20525470	hsa-miR-6754-3p	MIMAT0027409	1.9	1.52	0.28	0.22	1.3	0.030048	0.783209

20519425	hsa-miR-4646-5p	MIMAT0019707	2.07	1.68	0.46	0.26	1.3	0.037887	0.783209
20534241	HBII-296B	HBII-296B	1.74	1.36	0.45	0.1	1.3	0.045545	0.801082
20533544	ENSG00000238822	ENSG0000023{	1.42	1.05	0.14	0.07	1.29	0.000141	0.260306
20533805	ENSG00000251822	ENSG0000025{	1.21	0.85	0.16	0.09	1.29	0.00191	0.633418
20536467	hsa-mir-4257	MI0015856	1.25	0.89	0.28	0.15	1.29	0.032124	0.783209
20536802	hsa-mir-4522	MI0016889	1.31	0.94	0.27	0.24	1.29	0.033221	0.783209
20535911	hsa-mir-1282	MI0006429	1.95	1.58	0.43	0.45	1.29	0.039836	0.79565
20538105	SNORA38B	SNORA38B	1.64	1.28	0.13	0.29	1.28	0.015208	0.783209
20503897	hsa-miR-510-5p	MIMAT0002882	1.44	1.09	0.18	0.31	1.28	0.023043	0.783209
20519651	hsa-miR-4773	MIMAT0019928	1.78	1.43	0.18	0.3	1.28	0.029244	0.783209
20515539	hsa-miR-466	MIMAT0015002	1.66	1.31	0.26	0.16	1.27	0.015123	0.783209
20519670	hsa-miR-4783-3p	MIMAT0019947	1.48	1.13	0.23	0.26	1.27	0.027111	0.783209
20522042	hsa-miR-5705	MIMAT0022499	1.35	1	0.17	0.22	1.27	0.033385	0.783209
20526181	hsa-miR-7111-3p	MIMAT0028120	1.36	1.02	0.23	0.15	1.27	0.034348	0.783209
20518431	hsa-miR-3910	MIMAT0018184	2.35	2	0.59	0.55	1.27	0.04205	0.796329
20525611	hsa-miR-6825-5p	MIMAT0027550	1.56	1.22	0.39	0.16	1.27	0.044977	0.801082
20537012	hsa-mir-4770	MI0017411	1.24	0.91	0.16	0.21	1.26	0.009716	0.72094
20532802	ENSG00000200496	ENSG0000020(	1.38	1.05	0.19	0.24	1.26	0.028944	0.783209
20500140	hsa-miR-20a-3p	MIMAT0004493	1.54	1.2	0.26	0.29	1.26	0.030193	0.783209
20525503	hsa-miR-6771-5p	MIMAT0027442	5.49	5.16	0.21	0.33	1.26	0.031331	0.783209
20534242	HBII-296B	HBII-296B	1.73	1.4	0.4	0.09	1.26	0.037914	0.783209

20534510	hsa-mir-7-3	MI0000265	1.14	0.81	0.34	0.17	1.26	0.045229	0.801082
20533092	ENSG00000212445	ENSG0000021:	1.18	0.84	0.27	0.23	1.26	0.046838	0.802171
20538012	hsa-mir-8054	MI0025890	1.14	0.83	0.14	0.08	1.24	0.00452	0.64955
20537265	hsa-mir-6125	MI0021259	2.85	2.54	0.17	0.18	1.24	0.005627	0.687402
20505965	hsa-miR-509-3-5p	MIMAT0004975	1.34	1.03	0.27	0.18	1.24	0.010253	0.723245
20534749	hsa-mir-1-1	MI0000651	1.28	0.96	0.23	0.09	1.24	0.022943	0.783209
20525445	hsa-miR-6741-3p	MIMAT0027384	1.21	0.9	0.21	0.22	1.24	0.027578	0.783209
20506855	hsa-miR-1261	MIMAT0005913	1.39	1.08	0.26	0.2	1.24	0.031039	0.783209
20537506	hsa-mir-6763	MI0022608	1.4	1.1	0.11	0.19	1.23	0.004792	0.64955
20515513	hsa-miR-3119	MIMAT0014981	1.22	0.93	0.08	0.18	1.23	0.006075	0.687402
20532685	ACA49	ACA49	1.68	1.38	0.22	0.14	1.23	0.009966	0.72094
20500125	hsa-let-7f-2-3p	MIMAT0004487	1.05	0.75	0.11	0.19	1.23	0.018887	0.783209
20536977	hsa-mir-4739	MI0017377	1.04	0.74	0.14	0.22	1.23	0.022524	0.783209
20517753	hsa-miR-4330	MIMAT0016924	1.23	0.94	0.19	0.19	1.22	0.017875	0.783209
20525703	hsa-miR-6871-5p	MIMAT0027642	1.62	1.33	0.22	0.26	1.22	0.024419	0.783209
20519535	hsa-miR-4711-3p	MIMAT0019817	1.11	0.82	0.36	0.2	1.22	0.027174	0.783209
20533110	ENSG00000212551	ENSG0000021:	1.41	1.13	0.27	0.2	1.22	0.037472	0.783209
20533220	ENSG00000238329	ENSG0000023{	1.36	1.07	0.25	0.19	1.22	0.041922	0.796329
20523013	hsa-miR-6081	MIMAT0023706	1.09	0.81	0.19	0.17	1.22	0.045381	0.801082
20536985	hsa-mir-4746	MI0017385	1.13	0.85	0.11	0.17	1.21	0.00829	0.687402
20520209	hsa-miR-5006-3p	MIMAT0021034	1.31	1.04	0.34	0.2	1.21	0.027159	0.783209

20538206	U56	U56	8.4	8.13	0.19	0.2	1.21	0.040311	0.79565
20533432	ENSG00000238657	ENSG0000023€	1.2	0.94	0.1	0.11	1.2	0.004724	0.64955
20536306	hsa-mir-3188	MI0014232	1.12	0.86	0.24	0.12	1.2	0.008228	0.687402
20538245	U73b	U73b	1.43	1.17	0.21	0.1	1.2	0.018991	0.783209
20525627	hsa-miR-6833-5p	MIMAT0027566	1.58	1.31	0.25	0.11	1.2	0.034266	0.783209
20504568	hsa-miR-1323	MIMAT0005795	1.6	1.34	0.39	0.2	1.2	0.040382	0.79565
20537563	hsa-mir-6819	MI0022664	2.09	1.83	0.18	0.17	1.19	0.007447	0.687402
20519406	hsa-miR-4632-3p	MIMAT0019688	1.44	1.19	0.15	0.13	1.19	0.013517	0.783209
20519448	hsa-miR-4661-3p	MIMAT0019730	1.12	0.86	0.13	0.14	1.19	0.026639	0.783209
20518833	hsa-miR-4453	MIMAT0018975	1.12	0.87	0.15	0.11	1.19	0.032066	0.783209
20532877	ENSG00000201674	ENSG0000020	1.09	0.84	0.21	0.14	1.19	0.032775	0.783209
20534197	ENSG00000268305	ENSG0000026€	1.09	0.84	0.21	0.14	1.19	0.032775	0.783209
20500768	hsa-miR-126-5p	MIMAT0000444	1.23	0.97	0.46	0.19	1.19	0.032779	0.783209
20534314	HBII-85-21	HBII-85-21	1.32	1.07	0.39	0.3	1.19	0.042464	0.796329
20534361	hsa-mir-21	MI0000077	2.35	2.1	0.16	0.34	1.19	0.042887	0.801082
20518433	hsa-miR-3912-5p	MIMAT0027036	1.24	0.99	0.17	0.18	1.19	0.043654	0.801082
20517925	hsa-miR-3670	MIMAT0018093	1.05	0.79	0.15	0.15	1.19	0.047408	0.803999
20535243	hsa-mir-455	MI0003513	1.49	1.26	0.04	0.09	1.18	0.000361	0.260306
20535699	hsa-mir-297	MI0005775	1.17	0.93	0.11	0.14	1.18	0.0078	0.687402
20533016	ENSG00000207502	ENSG0000020	1.43	1.19	0.18	0.16	1.18	0.01345	0.783209
20504392	hsa-miR-639	MIMAT0003309	1.53	1.29	0.24	0.14	1.18	0.030223	0.783209

20538224	U68	U68	4.94	4.7	0.16	0.18	1.18	0.037574	0.783209
20500713	hsa-let-7g-5p	MIMAT0000414	11.73	11.49	0.3	0.24	1.18	0.044646	0.801082
20529144	hsa-miR-7852-3p	MIMAT0030427	1.63	1.39	0.4	0.25	1.18	0.04503	0.801082
20533991	ENSG00000252566	ENSG0000025[illegible]	1.19	0.96	0.13	0.09	1.17	0.01393	0.783209
20505741	hsa-miR-300	MIMAT0004903	1.21	0.98	0.15	0.14	1.17	0.024226	0.783209
20536800	hsa-mir-4521	MI0016887	1.53	1.31	0.19	0.12	1.17	0.027494	0.783209
20532821	ENSG00000200897	ENSG0000020[illegible]	1.16	0.93	0.16	0.18	1.17	0.028115	0.783209
20504401	hsa-miR-647	MIMAT0003317	0.96	0.73	0.18	0.13	1.17	0.036433	0.783209
20532626	ACA16	ACA16	1.98	1.76	0.22	0.23	1.17	0.044854	0.801082
20536704	hsa-mir-548ag-2	MI0016794	1.71	1.49	0.12	0.2	1.16	0.004452	0.64955
20536875	hsa-mir-4654	MI0017282	1.26	1.05	0.13	0.14	1.16	0.014733	0.783209
20521781	hsa-miR-4524b-5p	MIMAT0022255	1.31	1.1	0.21	0.08	1.16	0.017243	0.783209
20532989	ENSG00000207100	ENSG0000020[illegible]	1.38	1.17	0.25	0.12	1.16	0.018713	0.783209
20522026	hsa-miR-5692a	MIMAT0022484	1.02	0.8	0.09	0.18	1.16	0.03013	0.783209
20532752	ENSG00000199769	ENSG0000019[illegible]	1.17	0.95	0.18	0.14	1.16	0.037348	0.783209
20537133	hsa-mir-5191	MI0018170	1.27	1.05	0.19	0.11	1.16	0.038057	0.783705
20506832	hsa-miR-1243	MIMAT0005894	1.16	0.95	0.11	0.1	1.15	0.01022	0.723245
20536571	hsa-mir-3667	MI0016068	1.5	1.29	0.16	0.14	1.15	0.013639	0.783209
20520203	hsa-miR-5003-3p	MIMAT0021026	1.06	0.85	0.17	0.1	1.15	0.023978	0.783209
20506884	hsa-miR-1284	MIMAT0005941	1.12	0.91	0.13	0.14	1.15	0.026446	0.783209
20519572	hsa-miR-4729	MIMAT0019851	1.02	0.83	0.16	0.11	1.15	0.027115	0.783209

20504341	hsa-miR-601	MIMAT0003269	1.28	1.08	0.31	0.12	1.15	0.033486	0.783209
20525745	hsa-miR-6892-5p	MIMAT0027684	1.34	1.14	0.2	0.14	1.15	0.034963	0.783209
20534814	hsa-mir-200a	MI0000737	1.14	0.93	0.15	0.18	1.15	0.049695	0.814413
20536681	hsa-mir-548ad	MI0016770	1.24	1.06	0.08	0.15	1.14	0.007913	0.687402
20537236	hsa-mir-5739	MI0019412	1.05	0.86	0.08	0.15	1.14	0.014964	0.783209
20537871	hsa-mir-7155	MI0023615	1.09	0.9	0.19	0.11	1.14	0.020428	0.783209
20532672	ACA42	ACA42	3.27	3.08	0.18	0.21	1.14	0.029692	0.783209
20536499	hsa-mir-4278	MI0015888	1.13	0.94	0.13	0.13	1.14	0.033319	0.783209
20537455	hsa-mir-6715a	MI0022548	0.98	1.05	0.06	0.06	-1.05	0.013973	0.783209
20536925	hsa-mir-4694	MI0017327	0.97	1.04	0.09	0.09	-1.05	0.035453	0.783209
20536859	hsa-mir-4639	MI0017266	0.91	1.01	0.13	0.08	-1.07	0.030293	0.783209
20536486	hsa-mir-4269	MI0015875	0.81	0.94	0.11	0.19	-1.09	0.018897	0.783209
20537567	hsa-mir-6823	MI0022668	0.92	1.04	0.15	0.13	-1.09	0.023953	0.783209
20535343	hsa-mir-599	MI0003611	1.01	1.13	0.16	0.09	-1.09	0.028484	0.783209
20536551	hsa-mir-3646	MI0016046	1.14	1.27	0.12	0.09	-1.09	0.04128	0.795727
20519648	hsa-miR-4771	MIMAT0019925	1.02	1.16	0.11	0.15	-1.1	0.015249	0.783209
20519504	hsa-miR-4693-3p	MIMAT0019785	0.99	1.21	0.11	0.21	-1.17	0.036354	0.783209
20536631	hsa-mir-3925	MI0016433	0.81	1.04	0.16	0.15	-1.17	0.041599	0.796329
20504386	hsa-miR-633	MIMAT0003303	0.93	1.16	0.23	0.14	-1.17	0.047375	0.803999
20519410	hsa-miR-4635	MIMAT0019692	0.94	1.18	0.14	0.08	-1.18	0.002949	0.64955
20500478	hsa-miR-218-5p	MIMAT0000275	0.88	1.12	0.11	0.15	-1.18	0.010487	0.731987

20525009	hsa-miR-6504-5p	MIMAT0025464	0.76	1.01	0.16	0.24	-1.18	0.018393	0.783209
20538034	hsa-mir-8075	MI0025911	1.69	1.92	0.14	0.22	-1.18	0.025309	0.783209
20535850	hsa-mir-548f-1	MI0006374	1.06	1.3	0.12	0.24	-1.18	0.042385	0.796329
20537613	hsa-mir-6867	MI0022714	1.44	1.67	0.27	0.29	-1.18	0.042492	0.796329
20519623	hsa-miR-4756-3p	MIMAT0019900	0.83	1.08	0.12	0.11	-1.19	0.00343	0.64955
20519454	hsa-miR-4662b	MIMAT0019736	0.96	1.21	0.1	0.2	-1.19	0.005767	0.687402
20537136	hsa-mir-5194	MI0018173	0.89	1.14	0.15	0.12	-1.19	0.007481	0.687402
20536036	hsa-mir-2276	MI0011282	1.04	1.29	0.15	0.2	-1.19	0.020439	0.783209
20519621	hsa-miR-499b-3p	MIMAT0019898	1.03	1.28	0.1	0.23	-1.19	0.035402	0.783209
20534500	hsa-mir-30d	MI0000255	0.84	1.11	0.16	0.17	-1.2	0.014179	0.783209
20537060	hsa-mir-4802	MI0017450	0.78	1.1	0.12	0.14	-1.25	0.002215	0.638484
20536279	hsa-mir-3171	MI0014202	0.82	1.14	0.12	0.14	-1.25	0.007009	0.687402
20520217	hsa-miR-5009-3p	MIMAT0021042	0.97	1.67	0.23	0.81	-1.63	0.0396	0.79565
20525655	hsa-miR-6847-5p	MIMAT0027594	2.49	3.4	0.74	0.7	-1.87	0.025767	0.783209
20529134	hsa-miR-6516-5p	MIMAT0030417	3.41	4.47	0.22	0.55	-2.07	0.013613	0.783209
20500156	hsa-miR-27a-5p	MIMAT0004501	5.28	6.38	1.35	0.37	-2.14	0.039589	0.79565
20500145	hsa-miR-23a-5p	MIMAT0004496	5.6	6.78	0.51	0.4	-2.27	0.014511	0.783209
