# Supplementary material for: Genome-Wide MicroRNA Analysis of Peripheral Blood Mononuclear Cells Reveals Elevated miR-142-3p Expression as a Potential Biomarker for Secondary Syphilis
Source: Biomed Res Int. 2021 Jul 19;2021:5520053. doi: 10.1155/2021/5520053 (PMC8317471; doi:10.1155/2021/5520053)
Supplement: Supplementary 2 — Supplementary table 2: GO and KEGG enrichment analyses. [file 5520053.f2.pdf]

Supplementary table 2 GO and KEGG enrichment analysis.

Category	Term	Count	%	PValue	Genes	List Total	Pop Hits	Pop Total	Fold Enrichment	Bonferroni	Benjamini	FDR
GOTERM_BP_DIRECT	GO:0006351~transcription, DNA-templated	30	18.63	0.00	CCNT2, BCLAF1, ZNF676, ZNF367, ZEB2, NR3C1, ZEB1, NR2C1, PCGF3, ZNF682, PSIP1, NR2F6, PATZ1, GFI1, KDM5C, ATF7IP, KAT7, ZBTB20, RARG, ARID5B, ZBTB41, PPP1R10, ARNTL, RLF, BAZ1A, ATXN7, BNC2, JMJD1C, LCOR, RERE	149	1955	16792	1.73	0.96	0.80	5.08
GOTERM_BP_DIRECT	GO:0006355~regulation of transcription, DNA-templated	22	13.66	0.02	KAT7, RREB1, AFF4, ZBTB41, ZNF676, SOX5, NR3C1, AFF1, ARNTL, HMGA2, NR2C1, RLF, PCGF3, BAZ1A, ZNF682, BNC2, PSIP1, PATZ1, JMJD1C, GFI1, ERC1, LCOR	149	1504	16792	1.65	1.00	0.76	30.97
GOTERM_BP_DIRECT	GO:0045944~positive regulation of transcription from RNA polymerase II promoter	20	12.42	0.00	CCNT2, ARHGEF2, RARG, STRN3, ZEB2, NR3C1, ZEB1, ARNTL, HMGA2, NFATC2IP, RLF, PHIP, TET3, ATXN7, ASH1L, FOXF2, ARF4, PSIP1, TNKS RERE	149	981	16792	2.30	0.63	0.63	1.59
GOTERM_BP_DIRECT	GO:0000122~negative regulation of transcripti	14	8.70	0.01	ATF7IP, XPO1, ZBTB20, RARG, RREB1, STRN3, ARID5B, NR2F6, ZEB2, ZEB1, GFI1, HMGA2, RERE NR2C1	149	720	16792	2.19	1.00	0.75	16.31

GOTERM_BP_DIRECT	GO:0045892~negative regulation of transcriptio	12	7.45	0.00	ATF7IP, BCLAF1, EREG, STRN3, ARID5B, FOXF2, PATZ1, ARNTL, GFI1, ZEB1, HMGA2, KDM5C	149	499	16792	2.71	0.99	0.68	7.19
GOTERM_BP_DIRECT	GO:0006468~protein phosphorylation	9	5.59	0.05	MAP4K3, CCNT2, SGK1, TAOK1, ROCK2, MMD, WNK3, SIK1, MAP3K11	149	456	16792	2.22	1.00	0.85	54.68
GOTERM_BP_DIRECT	GO:0006366~transcription from RNA	9	5.59	0.08	CCNT2, RREB1, FOXF2, ASH1L, AFF4, SOX5, CREB5, NR3C1, HMGA2	149	513	16792	1.98	1.00	0.90	74.98
GOTERM_BP_DIRECT	GO:0007067~mitotic nuclear	8	4.97	0.01	BOD1, SNX18, CLTA, ARHGEF2, TNKS, NR3C1, HMGA2, EML4	149	248	16792	3.64	1.00	0.72	9.72
GOTERM_BP_DIRECT	GO:0015031~protein transport	8	4.97	0.06	TMEM115, EIF5AL1, ARF4, LRP1B, AAGAB, TNKS, ERC1, MYH9	149	395	16792	2.28	1.00	0.87	62.72
GOTERM_BP_DIRECT	GO:0006886~intracellular protein	7	4.35	0.02	XPO1, SNX18, CLTA, ARHGEF2, IPO7, STAM, NSF	149	236	16792	3.34	1.00	0.78	25.22
GOTERM_BP_DIRECT	GO:0043065~positive regulation	7	4.35	0.05	ARHGEF2, BCLAF1, RARG, MTCH1, RAC1, HMGA2, APC	149	300	16792	2.63	1.00	0.84	55.36
GOTERM_BP_DIRECT	GO:0051301~cell division	7	4.35	0.09	CCNT2, BOD1, CLTA, ARHGEF2, TNKS, NR3C1, HMGA2	149	350	16792	2.25	1.00	0.90	77.21
GOTERM_BP_DIRECT	GO:0007264~small GTPase mediated	6	3.73	0.07	ARF4, RAC1, RHEB, TRIM23, RAB1A, RHOBTB3	149	246	16792	2.75	1.00	0.87	66.34
GOTERM_BP_DIRECT	GO:0006897~endocyto	5	3.11	0.03	C9ORF72, SNX18, USP33, RAB1A, GHR	149	139	16792	4.05	1.00	0.80	42.55
GOTERM_BP_DIRECT	GO:0016192~vesicle-mediated	5	3.11	0.05	CLTA, VAMP3, NSF, RAB1A, RHOBTB3	149	152	16792	3.71	1.00	0.84	52.04

GOTERM_BP_DIRECT	GO:0006367~transcription initiation from RNA	5	3.11	0.05	RARG, NR2F6, TEAD1, NR3C1, NR2C1	149	152	16792	3.71	1.00	0.84	52.04
GOTERM_BP_DIREC	GO:0016477~cell	5	3.11	0.07	ITGAV, ARF4, USP33, RAB1A, APC	149	172	16792	3.28	1.00	0.87	65.77
GOTERM_BP_DIRECT	GO:0034446~substrate adhesion-dependent	4	2.48	0.00	ITGAV, RAC1, VAMP3, RAB1A	149	38	16792	11.86	0.99	0.77	6.88
GOTERM_BP_DIREC	GO:0051028~mRNA	4	2.48	0.01	XPO1, EIF5AL1, QKI, TNKS	149	47	16792	9.59	1.00	0.68	12.15
GOTERM_BP_DIRECT	GO:0050790~regulation of	4	2.48	0.02	SGK1, IPO7, ZEB2, MTMR9	149	66	16792	6.83	1.00	0.76	27.82
GOTERM_BP_DIREC	GO:0060021~palate	4	2.48	0.03	BNC2, ARID5B, FOXF2, TIPARP	149	76	16792	5.93	1.00	0.77	37.65
GOTERM_BP_DIRECT	GO:0006890~retrograde vesicle-mediated transport,	4	2.48	0.04	TMEM115, ARF4, NSF, RAB1A	149	82	16792	5.50	1.00	0.79	43.72
GOTERM_BP_DIRECT	GO:0001921~positive regulation	3	1.86	0.01	INPP5F, VAMP3, NSF	149	14	16792	24.15	1.00	0.66	9.83
GOTERM_BP_DIRECT	GO:0051298~centrosome	3	1.86	0.01	ROCK2, CEP192, USP33	149	19	16792	17.79	1.00	0.73	17.26
GOTERM_BP_DIRECT	GO:0006471~protein ADP-	3	1.86	0.01	ARF4, TIPARP, TNKS	149	21	16792	16.10	1.00	0.76	20.57
GOTERM_BP_DIRECT	GO:0022604~regulation of cell	3	1.86	0.02	PHIP, FMNL2, RAC1	149	25	16792	13.52	1.00	0.78	27.56

GOTERM_BP_DIRECT	GO:0045840~positive regulation of mitotic	3	1.86	0.02	PHIP, EREG, SH2B1	149	26	16792	13.00	1.00	0.76	29.36
GOTERM_BP_DIRECT	GO:2000145~regulation of cell	3	1.86	0.03	EREG, ROCK2, INPP5F	149	28	16792	12.07	1.00	0.77	32.99
GOTERM_BP_DIREC	GO:0000281~mitotic	3	1.86	0.03	SNX18, APC, MYH10	149	29	16792	11.66	1.00	0.75	34.82
GOTERM_BP_DIRECT	GO:0048008~platelet-derived growth factor	3	1.86	0.03	ARID5B, GAB1, TIPARP	149	29	16792	11.66	1.00	0.75	34.82
GOTERM_BP_DIRECT	GO:2001237~negative regulation of extrinsic apoptotic	3	1.86	0.04	PHIP, ITGAV, SGMS1	149	38	16792	8.90	1.00	0.84	50.85
GOTERM_BP_DIRECT	GO:0045860~positive regulation of protein	3	1.86	0.06	EREG, MMD, TAB2	149	47	16792	7.19	1.00	0.88	64.98
GOTERM_BP_DIRECT	GO:0043647~inositol phosphate metabolic	3	1.86	0.06	ITPKB, IPMK, INPP5A	149	47	16792	7.19	1.00	0.88	64.98
GOTERM_BP_DIREC	GO:0007254~JNK	3	1.86	0.07	MAP4K3, TAB2, MAP3K11	149	49	16792	6.90	1.00	0.87	67.75
GOTERM_BP_DIREC	GO:0048511~rhythmic	3	1.86	0.08	ROCK2, SIK1, KDM5C	149	54	16792	6.26	1.00	0.90	74.02
GOTERM_BP_DIRECT	GO:0007173~epidermal growth factor	3	1.86	0.09	EREG, ARF4, GAB1	149	56	16792	6.04	1.00	0.90	76.27

GOTERM_BP_DIRECT	GO:0043401~steroid hormone mediated	3	1.86	0.09	RARG, NR2F6, NR2C1	149	57	16792	5.93	1.00	0.89	77.34
GOTERM_BP_DIRECT	GO:0045732~positive regulation of protein	3	1.86	0.10	TIPARP, NSF, APC	149	60	16792	5.63	1.00	0.90	80.34
GOTERM_BP_DIRECT	GO:0044236~multicellular organism	2	1.24	0.02	TIPARP, GHR	149	2	16792	####	1.00	0.79	24.33
GOTERM_BP_DIRECT	GO:0006452~translational	2	1.24	0.03	EIF5AL1, EIF5A	149	3	16792	75.13	1.00	0.76	34.18
GOTERM_BP_DIRECT	GO:2000074~regulation of type B pancreatic	2	1.24	0.03	RHEB, ARNTL	149	3	16792	75.13	1.00	0.76	34.18
GOTERM_BP_DIRECT	GO:0045905~positive regulation of	2	1.24	0.03	EIF5AL1, EIF5A	149	3	16792	75.13	1.00	0.76	34.18
GOTERM_BP_DIRECT	GO:0042977~activation of JAK2	2	1.24	0.03	PRLR, GHR	149	3	16792	75.13	1.00	0.76	34.18
GOTERM_BP_DIRECT	GO:0045901~positive regulation of	2	1.24	0.03	EIF5AL1, EIF5A	149	4	16792	56.35	1.00	0.79	42.74
GOTERM_BP_DIREC	GO:0060613~fat pad	2	1.24	0.05	ARID5B, HMGA2	149	6	16792	37.57	1.00	0.84	56.67
GOTERM_BP_DIRECT	GO:0072383~plus-end-directed vesicle	2	1.24	0.06	KIF5B, FYCO1	149	7	16792	32.20	1.00	0.88	62.31

GOTERM_BP_DIRECT	GO:0021799~cerebral cortex radially	2	1.24	0.08	RAC1, FBXO45	149	9	16792	25.04	1.00	0.89	71.49
GOTERM_BP_DIRECT	GO:2001022~positive regulation of response	2	1.24	0.08	BCLAF1, HMGA2	149	10	16792	22.54	1.00	0.90	75.20
GOTERM_BP_DIRECT	GO:0048016~inositol phosphate-mediated	2	1.24	0.09	ITPR3, INPP5A	149	11	16792	20.49	1.00	0.90	78.43

GOTERM_CC_DIRECT	GO:0005634~nucleus	72	44.72	0.00	CCNT2, XPO1, C9ORF72, OSBP, CPEB2, FIGNL1, ZNF676, EIF5A, ITPKB, ZEB2, ZEB1, SGMS1, ACBD5, NFATC2IP, DIP2A, PCGF3, ZNF682, FOXF2, NR2F6, PSIP1, QKI, RBM47, PATZ1, SIK1, IPMK, KDM5C, MTUS1, RBMS1, GHR, ATF7IP, SGK1, ZBTB20, RARG, RREB1, ROCK2, ARID5B, STRN3, MBD6, BTBD7, ARNTL, HMGA2, MYH9, BRWD3, ASH1L, JMJD1C, LCOR, BCLAF1, TIPARP, SOX5, ZNF367, NR3C1, NR2C1, PHIP, RAC1, SH2B1, GFI1, TSEN34, HECTD1, APC, KAT7, PELI1, ZBTB41, PPP1R10, CREB5, TRIM23, TMEM115, RLF, TET3, ATXN7, BNC2, RERE, MYH10	153	5415	18224	1.58	0.00	0.00	0.01

GOTERM_CC_DIRECT	GO:0005737~cytoplasm	55	34.16	0.04	XPO1, C9ORF72, OSBP, CPEB2, FIGNL1, UTRN, EIF5A, ZEB1, NFATC2IP, AAGAB, QKI, SIK1, NSF, ATF7IP, ARHGEF2, SGK1, TWF1, KIF5B, C6ORF47, RREB1, STRN3, WNK3, ARNTL, PFKM, MYH9, EML4, MAP4K3, CCDC6, FAM114A1, IPO7, ASH1L, PGM1, ERC1, MAP3K11, BCLAF1, NR3C1, PDSS2, STAU1, AKT1S1, RAC1, MTMR9, FBXO3, USP33, APC, HECTD1, BOD1, KAT7, CEP192, TAOK1, ITPR3, RASSF3, TET3, ATXN7, BNC2, MYH10	153	5222	18224	1.25	1.00	0.43	39.61
GOTERM_CC_DIRECT	GO:0005654~nucleoplasm	43	26.71	0.00	CCNT2, XPO1, SNX18, C9ORF72, BCLAF1, OSBP, UTRN, ZNF367, NR3C1, ZEB1, SMUG1, ACBD5, NR2C1, AKT1S1, AAGAB, PSIP1, NR2F6, PATZ1, FBXO3, IPMK, USP33, KDM5C, TSEN34, APC, ATF7IP, ZBTB20, SGK1, KAT7, RARG, RREB1, STRN3, ARID5B, MBD6, PPP1R10, TEAD1, ARNTL, ITPR3, HMGA2, IPO7, ATXN7, ASH1L, JMJD1C, EP400	153	2784	18224	1.84	0.01	0.01	0.08

GOTERM_CC_DIRECT	GO:0005829~cytosol	42	26.09	0.00	XPO1, CLTA, OSBP, EIF5A, ITPKB, RAB1A, ARHGAP12, TAGAP, AKT1S1, GAB1, RAC1, PUM1, AAGAB, PSIP1, TNKS, STAM, SH2B1, FBXO3, PIK3R6, FNDC3A, NSF, RHOBTB3, APC, FMNL2, SGK1, PELI1, ARHGEF2, OSBPL3, TAOK1, CEP192, ROCK2, WNK3, PFKM, MARK3, MYH9, TAB2, IPO7, ARF4, PGM1, RHEB, VAMP3, MYH10	153	3315	18224	1.51	0.66	0.14	5.83
GOTERM_CC_DIRECT	GO:0016020~membrane	30	18.63	0.01	XPO1, CLTA, OSBP, MMGT1, UTRN, MMD, EIF5A, ITPKB, SGMS1, ACBD5, STAU1, ITGAV, RAC1, MTCH1, PIK3R6, SH2B1, FYCO1, FNDC3A, INPP5A, MGAT4A, OSBPL3, KIF5B, MYH9, ITPR3, EML4, ATP2A2, IPO7, ARF4, RHEB, MAP3K11	153	2200	18224	1.62	0.85	0.21	9.93
GOTERM_CC_DIRECT	GO:0005622~intracellular	23	14.29	0.00	XPO1, TWF1, ARHGEF2, ROCK2, ZNF676, RAB1A, MAP4K3, ZNF682, EREG, IPO7, ITGAV, MTCH1, RAC1, ARF4, RHEB, VAMP3, STAM, JMJD1C, SH2B1, INPP5A, GHR, MAP3K11, RHOBTB3	153	1332	18224	2.06	0.31	0.07	2.01

GOTERM_CC_DIRECT	GO:0000139~Golgi membrane	14	8.70	0.00	MGAT4A, OSBP, MMGT1, SGMS1, TRIM23, RAB1A, TMEM115, TMEM59, RAC1, RHEB, TNKS, ERC1, FNDC3A, NSF	153	591	18224	2.82	0.27	0.08	1.75
GOTERM_CC_DIRECT	GO:0005794~Golgi apparatus	14	8.70	0.03	ARHGEF2, OSBP, MMGT1, STRN3, MMD, RAB1A, TMEM115, PLA2G12A, ASH1L, ARF4, TNKS, FNDC3A, USP33, MTUS1	153	863	18224	1.93	1.00	0.36	31.05
GOTERM_CC_DIRECT	GO:0043025~neuronal cell body	8	4.97	0.02	ALCAM, ARHGEF2, STRN3, INPP5F, ITPR3, GHR, STAU1, MYH10	153	315	18224	3.03	0.98	0.30	19.36
GOTERM_CC_DIRECT	GO:0005874~microtubule	7	4.35	0.05	RASSF3, ARHGEF2, KIF5B, MTUS1, EML4, MAP3K11, APC	153	311	18224	2.68	1.00	0.45	45.58
GOTERM_CC_DIRECT	GO:0005913~cell-cell adherens	7	4.35	0.05	FMNL2, TWF1, KIF5B, ERC1, MYH9, RAB1A, APC	153	323	18224	2.58	1.00	0.49	50.81
GOTERM_CC_DIRECT	GO:0032587~ruffle membrane	6	3.73	0.00	TWF1, ARHGEF2, ITGAV, ARF4, RAC1, APC	153	82	18224	8.72	0.13	0.05	0.79
GOTERM_CC_DIRECT	GO:0005819~spindle	6	3.73	0.00	CLTA, ARHGEF2, NR3C1, MYH9, MTUS1, MYH10	153	121	18224	5.91	0.55	0.12	4.35
GOTERM_CC_DIRECT	GO:0005923~bicellular tight	5	3.11	0.01	ARHGEF2, ASH1L, WNK3, CLDN12, APC	153	113	18224	5.27	0.97	0.29	17.46
GOTERM_CC_DIRECT	GO:0005667~transcription factor	5	3.11	0.08	ATF7IP, RARG, FOXF2, ARNTL, ZEB1	153	193	18224	3.09	1.00	0.54	64.85
GOTERM_CC_DIREC	GO:0005643~nuclear	4	2.48	0.02	IPO7, EIF5AL1, EIF5A, TNKS	153	72	18224	6.62	0.99	0.33	25.13

GOTERM_CC_DIRECT	GO:0031901~early endosome	4	2.48	0.07	MMGT1, RAC1, INPP5F, STAM	153	116	18224	4.11	1.00	0.55	62.19
GOTERM_CC_DIREC	GO:0000242~pericentr	3	1.86	0.01	CEP192, RAC1, TNKS	153	19	18224	18.81	0.92	0.24	12.96
GOTERM_CC_DIRECT	GO:0036464~cytoplasmic ribonucleop	3	1.86	0.02	ROCK2, RAC1, STAU1	153	25	18224	14.29	0.99	0.30	21.07
GOTERM_CC_DIRECT	GO:0031594~neuromuscular	3	1.86	0.08	UTRN, MYH9, MYH10	153	55	18224	6.50	1.00	0.55	64.23
GOTERM_CC_DIREC	GO:0005903~brush	3	1.86	0.09	MYH9, ITPR3, MYH10	153	60	18224	5.96	1.00	0.58	69.92
GOTERM_CC_DIREC	GO:0005776~autopha	3	1.86	0.10	C9ORF72, FYCO1, USP33	153	64	18224	5.58	1.00	0.59	74.00
GOTERM_CC_DIREC	GO:0097513~myosin II	2	1.24	0.02	MYH9, MYH10	153	3	18224	79.41	1.00	0.34	27.54
GOTERM_CC_DIREC	GO:0000138~Golgi	2	1.24	0.04	TMEM59, SGMS1	153	5	18224	47.64	1.00	0.43	41.55
GOTERM_CC_DIREC	GO:0005642~annulate	2	1.24	0.04	XPO1, EIF5A	153	5	18224	47.64	1.00	0.43	41.55
GOTERM_CC_DIREC	GO:0016460~myosin II	2	1.24	0.06	MYH9, MYH10	153	7	18224	34.03	1.00	0.49	52.85
GOTERM_CC_DIRECT	GO:0031095~platelet dense tubular	2	1.24	0.07	ATP2A2, ITPR3	153	9	18224	26.47	1.00	0.56	61.96
GOTERM_CC_DIREC	GO:0042641~actomyo	2	1.24	0.10	MYH9, MYH10	153	12	18224	19.85	1.00	0.59	72.44

GOTERM_MF_DIRECT	GO:0005515~protein binding	110	68.32	0.00	XPO1, UTRN, EIF5A, ITPKB, RAB1A, GAB1, PUM1, AAGAB, PSIP1, STAM, ELOVL7, RBMS1, FLVCR1, PPP1R37, RPUSD4, RARG, KIF5B, ROCK2, STRN3, WNK3, SYNJ2BP, ARNTL, MYH9, MARK3, MAP4K3, CCDC6, BAZ1A, PGM1, VAMP3, ERC1, EP400, SOX5, NR2C1, ALCAM, AKT1S1, ITGAV, MTCH1, RAC1, TNKS, FBXO3, FBXO45, HECTD1, RHOBTB3, KAT7, PELI1, OSBPL3, TAOK1, TEAD1, TMEM115, TET3, ATP2A2, ARF4, RERE, MYH10, CCNT2, C9ORF72, CLTA, OSBP, FIGNL1, ZEB2, ZEB1, DIP2A, PCGF3, TMEM59, NR2F6, QKI, SIK1, NSF, GHR, ATF7IP, SGK1, ARHGEF2, ARID5B, PFKM, HMGA2, FAM114A1, EREG, IPO7, JMJD1C, LCOR, MAP3K11, SNX18, BCLAF1, NR3C1, SMUG1, STAU1, PHIP, INPP5F, MTMR9, SH2B1, PIK3R6, GFI1, FYCO1, MANBAL, USP33, INPP5A, APC, CEP192, AFF4, CREB5, AFF1, TRIM23, ITPR3, TAB2, RASSF3, RLF, PPP1R2, PRLR, ATXN7, TMTC3	149	8785	16881	1.42	0.00	0.00	0.00

GOTERM_MF_DIRECT	GO:0003677~DNA binding	28	17.39	0.00	BCLAF1, SOX5, ZNF367, ZEB2, NR3C1, ZEB1, SMUG1, NR2C1, FOXF2, NR2F6, PATZ1, KDM5C, ZBTB20, RARG, ARID5B, MBD6, ZBTB41, PPP1R10, TEAD1, ARNTL, HMGA2, RLF, TET3, BNC2, ASH1L, LCOR, EP400, RERE	149	1674	16881	1.90	0.34	0.19	1.76
GOTERM_MF_DIRECT	GO:0046872~metal ion binding	27	16.77	0.04	TIPARP, ZNF676, ZNF367, ZEB2, ZEB1, PCGF3, ZNF682, ITGAV, PATZ1, GFI1, FYCO1, NSF, HECTD1, MGAT4A, ARHGEF2, ZBTB20, RREB1, ROCK2, ZBTB41, PPP1R10, CREB5, PFKM, PRLR, ATP2A2, BNC2, RHEB, JMJD1C	149	2069	16881	1.48	1.00	0.62	39.45
GOTERM_MF_DIRECT	GO:0008270~zinc ion binding	20	12.42	0.01	ARHGEF2, KAT7, RARG, UTRN, NR3C1, ZEB1, TRIM23, TAB2, NR2C1, RLF, TET3, PCGF3, BAZ1A, BNC2, ASH1L, NR2F6, TNKS, USP33, KDM5C, RERE	149	1169	16881	1.94	0.88	0.50	8.63
GOTERM_MF_DIRECT	GO:0005524~ATP binding	20	12.42	0.06	TWF1, SGK1, KIF5B, ROCK2, FIGNL1, TAOK1, ITPKB, WNK3, PFKM, MYH9, MARK3, MAP4K3, ATP2A2, SIK1, IPMK, NSF, EP400, MAP3K11, MYH10, RHOBTB3	149	1495	16881	1.52	1.00	0.72	58.90

GOTERM_MF_DIRECT	GO:0003700~transcription factor activity, sequence-specific	17	10.56	0.01	KAT7, RARG, RREB1, STRN3, AFF4, SOX5, TEAD1, ZNF367, CREB5, NR3C1, AFF1, ARNTL, ZEB1, NR2C1, FOXF2, NR2F6, RERE	149	961	16881	2.00	0.96	0.55	12.85
GOTERM_MF_DIRECT	GO:0003676~nucleic acid binding	14	8.70	0.09	ZBTB20, CPEB2, RREB1, ZNF676, ZBTB41, CREB5, ZEB2, ZEB1, ZNF682, PATZ1, RBM47, GFI1, TSEN34, RBMS1	149	985	16881	1.61	1.00	0.78	72.62
GOTERM_MF_DIRECT	GO:0004674~protein serine/threonine kinase	9	5.59	0.02	MAP4K3, CCNT2, SGK1, TAOK1, ROCK2, WNK3, MARK3, SIK1, MAP3K11	149	376	16881	2.71	1.00	0.55	21.55
GOTERM_MF_DIRECT	GO:0003682~chromatin binding	9	5.59	0.02	CCNT2, ATXN7, ASH1L, PSIP1, MBD6, PATZ1, ZEB1, EP400, RERE	149	391	16881	2.61	1.00	0.58	25.87
GOTERM_MF_DIRECT	GO:0019901~protein kinase	8	4.97	0.05	CCNT2, UTRN, RAC1, RHEB, SIK1, NSF, GHR, APC	149	376	16881	2.41	1.00	0.69	48.32
GOTERM_MF_DIRECT	GO:0003924~GTPase activity	6	3.73	0.06	ARF4, RAC1, RHEB, TRIM23, RAB1A, MMAA	149	234	16881	2.91	1.00	0.72	53.43
GOTERM_MF_DIRECT	GO:0016301~kinase activity	6	3.73	0.06	MAP4K3, TAOK1, SGMS1, PIK3R6, TAB2, MAP3K11	149	241	16881	2.82	1.00	0.73	57.19
GOTERM_MF_DIRECT	GO:0017137~Rab	5	3.11	0.03	C9ORF72, RAC1, ERC1, NSF, RHOBTB3	149	134	16881	4.23	1.00	0.62	33.90
GOTERM_MF_DIRECT	GO:0003707~steroid hormone	4	2.48	0.01	RARG, NR2F6, NR3C1, NR2C1	149	56	16881	8.09	0.98	0.56	16.16

GOTERM_MF_DIRECT	GO:0001227~transcriptional repressor activity, RNA polymerase II	4	2.48	0.02	ARID5B, NR2F6, ZEB2, ZEB1	149	59	16881	7.68	0.99	0.54	18.38
GOTERM_MF_DIRECT	GO:0001078~transcriptional repressor activity, RNA polymerase II core	4	2.48	0.07	ZBTB20, GFI1, HMGA2, NR2C1	149	111	16881	4.08	1.00	0.73	64.39
GOTERM_MF_DIREC	GO:0017048~Rho	3	1.86	0.02	FMNL2, ARHGEF2, ROCK2	149	28	16881	12.14	1.00	0.58	28.69
GOTERM_MF_DIRECT	GO:0004864~protein phosphatas	3	1.86	0.03	PPP1R37, PPP1R2, PPP1R10	149	33	16881	10.30	1.00	0.62	36.95
GOTERM_MF_DIREC	GO:0043022~ribosom	3	1.86	0.07	CPEB2, EIF5AL1, EIF5A	149	51	16881	6.66	1.00	0.75	64.22
GOTERM_MF_DIRECT	GO:0008440~inositol-1,4,5-trisphospha	2	1.24	0.08	ITPKB, IPMK	149	10	16881	22.66	1.00	0.76	69.36
KEGG_PATHWAY	hsa04151:PI3K-Akt signaling	7	4.35	0.06	SGK1, PRLR, ITGAV, RAC1, RHEB, CREB5, GHR	57	345	6879	2.45	1.00	0.95	52.23
KEGG_PATHWAY	hsa05205:Proteoglycans	5	3.11	0.08	ROCK2, ITGAV, GAB1, RAC1, ITPR3	57	200	6879	3.02	1.00	0.91	62.52
KEGG_PATHWAY	hsa04972:Pancreatic	4	2.48	0.04	ATP2A2, PLA2G12A, RAC1, ITPR3	57	93	6879	5.19	1.00	1.00	38.23
KEGG_PATHWAY	hsa04070:Phosphatidylinositol	4	2.48	0.05	ITPKB, ITPR3, IPMK, INPP5A	57	98	6879	4.93	1.00	0.97	42.34

KEGG_PATHWAY	hsa04152:AMPK	4	2.48	0.08	AKT1S1, RHEB, CREB5, PFKM	57	123	6879	3.92	1.00	0.95	62.05
KEGG_PATHWAY	hsa04728:Dopaminergi	4	2.48	0.09	KIF5B, CREB5, ARNTL, ITPR3	57	128	6879	3.77	1.00	0.89	65.61
